# More than an outcome: a person-centered, ecological framework for eating disorder recovery

**DOI:** 10.1186/s40337-023-00768-1

**Published:** 2023-03-22

**Authors:** Therese E. Kenny, Stephen P. Lewis

**Affiliations:** Department of Psychology, University of Guelph, 50 Stone Road E., Guelph, ON N1G 2W1 Canada

**Keywords:** Eating disorder, Recovery, Lived experience, Framework

## Abstract

**Background:**

Eating disorder recovery is a complex phenomenon. While historical understandings focused on weight and behaviours, the importance of psychological factors is now widely recognized. It is also generally accepted that recovery is a non-linear process and is impacted by external factors. Recent research suggests a significant impact of systems of oppression, though these have not yet been named in models of recovery.

**Body:**

In this paper, we propose a research-informed, person-centered, and ecological framework of recovery. We suggest that there are two foundational tenets of recovery which apply broadly across experiences: recovery is non-linear and ongoing and there is no one way to do recovery. In the context of these tenets, our framework considers individual changes in recovery as determined by and dependent on external/personal factors and broader systems of privilege. Recovery cannot be determined by looking solely at an individual’s level of functioning; one must also consider the broader context of their life in which changes are being made. To conclude, we describe the applicability of the proposed framework and offer practical considerations for incorporating this framework in research, clinical, and advocacy settings.

**Supplementary Information:**

The online version contains supplementary material available at 10.1186/s40337-023-00768-1.

## Abstract (summary)

Eating disorder recovery research suggests that this is a complicated phenomenon that is characterized by psychological changes and impacted by external factors. Here, we present a novel framework for thinking about recovery which considers outcomes as a non-linear process and which occur in the broader context of someone’s life. Consistent with current views of recovery, we suggest that there is no one way to do recovery and that it is an ongoing journey. The framework also names that achieving certain recovery outcomes is dependent on (and should be considered in the context of) broader life experiences. This includes personal factors such as daily life experiences and other mental health challenges, as well as systems of oppression (e.g., White supremacy) which make recovery more attainable for some individuals. Importantly, we suggest that outcomes which have historically been included in recovery definitions (e.g., weight) may not be important to all individuals. Because of this, our framework is person-centered: individuals are able to determine what outcomes are important in their own recovery journey. We give some examples of how this framework may be useful in research, clinical, and advocacy settings.

**Supplementary Information:**

The online version contains supplementary material available at 10.1186/s40337-023-00768-1.

## Background

Researchers have examined eating disorder (ED) recovery using diverse methodological approaches [1], which has yielded a complex and nuanced body of research. While early conceptualizations of recovery focused on physical and behavioural markers such as weight gain and behavioural abstinence (e.g., [2]), the importance of psychological factors in recovery is now widely accepted [3]. In a qualitative meta-synthesis, de Vos and colleagues [4] identified psychological well-being as a central criterion for ED recovery [4]. Bardone-Cone and colleagues have published a series of studies which demonstrated that individuals who experience weight restoration and have not engaged in ED behaviours (i.e., binge eating, purging, fasting) over the past three months can be meaningfully distinguished by their scores on a measure of ED psychopathology. Specifically, individuals who reported scores within one standard deviation of community norms looked similar to individuals who have never had an ED on several measures (e.g., perfectionism, negative affect), while those with scores greater than one standard deviation above the mean looked more like individuals with a current ED on these same measures [5–15]. Research looking at clinician and lived experience definitions of recovery has found that these groups emphasize the importance of psychosocial factors, such as quality of life, relationship with food and body, and social connection (e.g., [16–20]).

While ‘recovery criteria’ can make it seem like recovery is a static state, individuals with lived ED experience frequently refer to recovery as an ongoing phenomenon. Notably, individuals who have had an ED consistently describe recovery as a process [21–24], and several studies have examined this process, noting common transitions and steps along the way [25–34]. Individuals with lived experience indicate that recovery is non-linear and contains ups and downs in motivation, and ED-related behaviours and thoughts [16, 21–24, 35, 36]. LaMarre and Rice [35] discuss how Western conceptualizations of time delineation may force individuals into describing their recovery in a linear way (i.e., this happened first, this happened next), when this may not reflect their experience [35]. The fuzzy boundaries between illness and ‘wellness’ make it challenging to delineate when someone enters recovery or illness [35] and challenge traditional notions of an illness-recovery-relapse timeline.

Another line of research has identified facilitators and barriers to recovery. This evidence suggests that social support and formal treatment may be important in recovery efforts (e.g., [17, 23, 30, 37]), pointing to the importance of external factors. Recovery takes place in the context of one’s broader life and will inevitably be impacted by external events [16]. For example, the COVID-19 pandemic has resulted in increasing ED symptoms and hospital admissions [38]. Changes in food insecurity, which may be linked to rising food costs (e.g., [39]), have also been associated with increased ED symptoms [40]. Recovery, therefore, does not exist in a vacuum, and although this is commonly appreciated by researchers and clinicians, it is not always explicitly stated in the recovery literature.

Extending from this, there has been a recent push to better understand how broader systemic factors impact EDs and recovery. Systems of oppression are systemic and directional power relationships in which one social group directly benefits at the expense of another [41]. Nixon [42] talks about these systems as the two sides of a coin; the same system which benefits one group, marginalizes another [42]. There is evidence that systems of oppression result in disparities in diagnosis and treatment for individuals with EDs. One study found that female (vs. male), White (vs. non-White), and underweight (vs. normal and higher weight) individuals were more likely to receive an ED diagnosis [43]. Individuals who were female, underweight, and from affluent socioeconomic backgrounds were also more likely to have accessed treatment in the past year [43]. Such inequities seem to be perpetuated by stereotypes about who gets EDs [44] and a lack of diversity in the field more generally [45]. Ultimately, this results in greater recovery promoting supports for privileged groups [21] and suggests the importance of identifying and naming sociopolitical factors in the recovery process [46].

A subset of studies has identified dominant messages about weight, bodies, and food as particularly impactful in ED recovery [16, 21, 44, 47, 48]. LaMarre and Rice [44] discuss how dominant biopedagogies in Western societies frame weight loss as an indicator of health/well-being [4]. Societal messages present weight gain as bad and moralize food and exercise [49]. Individuals navigating ED recovery must then act in ways that are counter to dominant cultural messages [44], which can make sustained recovery difficult [21, 47].

It seems then, that historical definitions of recovery which do not explicitly acknowledge external and systemic factors do not capture the complexity of the recovery process. Studies asking participants about their views on extant recovery definitions have found that individual outcome-based definitions often do not resonate with individuals with lived experience (e.g., [50, 51]). This suggests the need for a broader framework which incorporates process factors and situates recovery in the context of one’s unique lived experiences.

**Fig. 1:**
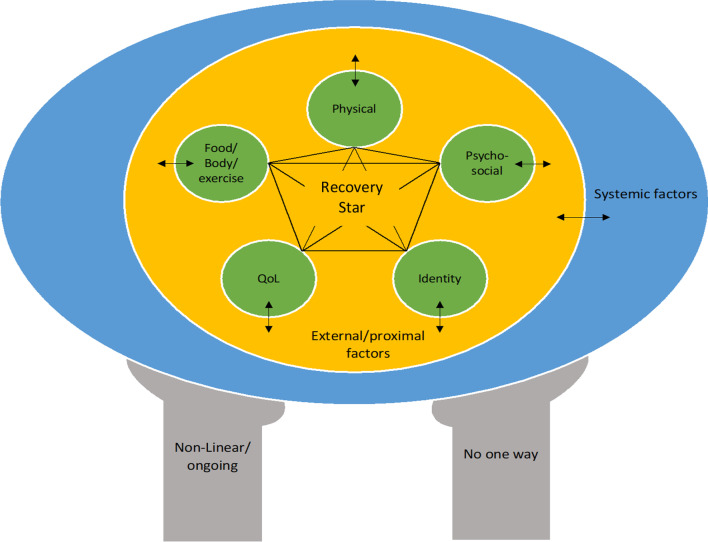
Visual representation of the proposed, person-centered, ecological model of recovery. Notes: QoL = quality of life; recovery star = the recovery star represents the constellation of changes that may occur over the course of recovery. This may include more conventional changes such as weight restoration and behavioural abstinence, as well as personal changes noted by the individual. We have included five possible changes which were noted by participants in our research [21] as examples. These are not fixed and can be changed, switched, or dropped as individuals see fit; external/proximal factors = external/proximal factors refer to factors in an individual’s environment which may impact their recovery journey, including their identified outcomes in the recovery star. These are factors that impact an individual at the individual level. They may be internal (e.g., mental health challenges) or external (e.g., death of a loved one) and changeable (e.g., medication adherence) or unchangeable (e.g., global pandemic); systemic factors = systemic factors refer to systems which may create inequities in recovery. This includes, but is not limited to, White supremacy, anti-fat bias, ableism, cissexism, and heteronormativity. These systems create power structures such that some individuals are granted easier access to diagnosis and treatment, while others are limited in their capacity to find support

In what follows, we present a novel person-centered recovery framework (Fig. 1) which aims to integrate and extend our understanding of ED recovery. From this perspective, we view recovery as an individualized and personal experience. What recovery ‘is’ depends on: (1) treatment approach and assumptions about aetiology in clinical practice [52]; (2) epistemological and ontological stances in research [1]; and (3) the multi-faceted lived experiences of individuals navigating recovery. This is different from clinical remission which focuses on the presence or absence of symptoms. We suggest that the latter may have more utility in determining treatment planning and patient readiness. Though there may be overlap between the two, as will be discussed later, clinical remission is neither necessary, nor sufficient for recovery according to this framework.

## Recovery tenets

The proposed framework is built upon two tenets. Although these are likely intuitive for individuals working in the field, we note them here as explicit parts of the framework to highlight their importance. These tenets are: (1) there is no one way to do recovery and (2) recovery is an ongoing and non-linear process. We view these as being the foundation of any recovery process and essential in situating one’s lived experiences.

### There is no one way to do ‘recovery’

Individuals with lived experience have shared diverse recovery experiences and explicitly noted the individuality of the recovery process [16, 21]. Participants in our and others’ research have emphasised that recovery is not ‘one-size-fits-all’ (e.g., [16, 35]), noting that what ‘recovery’ constitutes will differ across individuals [21]. For instance, some individuals may view symptom abatement as central to ED recovery, while others may focus on quality of life irrespective of symptom status. Additionally, individuals may choose to approach recovery in different ways, which may or may not include traditional treatment routes [53]. Even within treatment, recovery journeys can be different, suggesting that the recovery journey is flexible [50]. Recent research also points to challenges with recovery language. The term ‘recovery’ may invoke assumptions or connotations which are unhelpful in the recovery process [35, 47, 54, 55], and individuals with lived experience report feeling pressured to use the term ‘recovery’ when this may not be their preferred term [56]. Research from our lab suggests that individuals may prefer terms that reflect an ongoing process (e.g., journey, process, healing), absence of symptoms (e.g., remission), and/or a state of well-being (e.g., health, wellness) [56]. These are by no means exhaustive nor universal but do point to alternative terms which may resonate with individuals navigating ED recovery. Ultimately, this tenet reflects that the recovery process, including treatment seeking and language use, is unique for individuals with EDs.

## Recovery is an ongoing process with ups and downs

Several qualitative studies position ED recovery as a non-linear process [16, 21–24, 36]. In contrast to outcome-based definitions which tend to categorize recovery based on an individual’s functioning at a single time point (e.g., absence of behaviours and EDE-Q score < 1SD above community norms) (e.g., [5]), we suggest that there is merit to viewing recovery as an ongoing process which changes over time and to having this conversation with individuals early in the treatment process. While some individuals may view a return of symptoms (what some may term relapse) as distinct from the recovery process, participants in our and other research have noted that they view this as part of recovery (e.g., [21, 35, 50]). We therefore, use ongoing process here to describe the recovery journey more broadly, including times of progress and setback.

According to this tenet, model components will look different at different points for each individual’s recovery. This allows for flexibility in understanding where an individual is with respect to their current life circumstances. Importantly, participants in a study conducted by our research group talked about the ongoing nature of recovery even after treatment has terminated or they have reached a state of well-being [21]. For instance, participants shared challenges navigating ongoing body changes such as aging or pregnancy long after the ED had subsided. This tenet therefore, highlights the possibility of navigating thoughts, urges, or actions after the ED is ‘gone.’ From this perspective, recovery is an ongoing process which may extend into one’s broader life.

## The recovery star

At the center of the framework are recovery outcomes, including those typically used in other recovery definitions (i.e., weight, behaviours, cognitions). We have termed this the ‘recovery star’ to capture the constellation of changes that may transpire throughout one’s recovery journey. In the current paper, we present elements noted by individuals in a recent study in our lab (i.e., [21]) because these seemed to be comprehensive and consistent with past research (e.g., [4, 17, 22, 44, 57]). These are presented as starting points for individuals to reflect on in their journey and are not necessary, nor sufficient. These domains may not be relevant for all individuals, and individuals can personalise the number of points on their star to include more or fewer domains. Because of *the ongoing and non-linear nature* of the recovery process, the nature and number of domains may also shift over time. This notwithstanding, we briefly describe the five domains below.

## Physical stability

Whereas previous definitions of recovery use weight or resumption of menses as indicators of recovery (e.g., [2, 5, 58]), we have included a broader physical domain, which accounts for weight restoration as well as other indicators of physical well-being. For example, we have previously suggested that medical indicators of stability (e.g., cardiac functioning, bone health) may be better indicators of physical stability [59]. Individuals with lived experience have noted that physical stability also encompasses having adequate energy, improved cognitive functioning, and being present in one’s daily life [21, 60]. Physical stability here represents a broader range of indicators than just weight.

## Relationship with food, body, and exercise

Similar to *Physical stability*, this domain extends what has previously been included in recovery definitions. Traditionally, definitions include a measure of symptom abstinence (e.g., no binge eating, purging, fasting in the past three months) [5], which individuals with lived experience have indicated is not comprehensive, nor realistic [50]. Body image is implicitly included as part of ED psychopathology; exercise is not typically included in definitions of recovery though many professionals consider its importance in treatment settings. Individuals with lived experience note however, that shifts in one’s relationship with food, body, and exercise are all important in recovery [21]. Individuals with lived experience have specifically indicated that recovery involves the development of a freer and more flexible relationship with food and exercise, as well as working toward body acceptance [18, 19, 21]. Participants in our research point to having a relationship with food, body, and exercise which was relaxed and not impacting other areas of functioning [21]. Drawing on our framework’s tenets, this will naturally vary for different people at different points in their journey, which leaves flexibility in defining this domain.

## Psychosocial functioning

Individuals with lived experience have shared that recovery extends beyond weight and food. Notably, they underscore the importance of social connection (e.g., [16, 17, 19, 20]) and emotional and/or psychological well-being (e.g., [4, 18, 23]), including the development of alternative coping strategies [21]. Thus, we have included a psychosocial domain to encompass an individual’s capacity for social and emotional well-being. Again, what this comprises will differ across individuals but broadly it asks whether individuals believe they are engaged in positive social relationships and can experience their emotions effectively.

## Quality of life

It has been argued that there is a need for an ED-specific measure of quality of life in ED recovery [3]. Individuals with lived experience generally agree with the inclusion of a quality-of-life measure but talk about this as a broad construct, including one’s capacity to function in life [4, 21]. This latter point has been described in other models of recovery as ‘functional recovery,’ the capacity to function effectively in one’s life (e.g., holding a job, securing housing) [61]. We suggest that one element of ED recovery may then be quality of life which includes functional recovery, as well as one’s satisfaction with the life they are living. Again, this is flexible and will invariably look different for different individuals. The key take-away is that recovery for many people involves some level of life satisfaction and happiness, including capacity to engage in daily life. Having said this, we acknowledge that levels of functioning may differ depending on individuals’ circumstances and capacities. We caution readers in viewing functional recovery as the capacity to live alone or to hold a job, as this is inherently ableist. We suggest instead that this refer to an individual’s ability to function in their life in the way that they would like, acknowledging that there may be other factors which limit an individual’s capacity to do this.

## Identity

Finally, individuals with lived experience point to the role of identity in recovery. In a study of recovered professionals, Bowlby et al. [22] suggested that this involves de-identifying with the ED and finding a sense of meaning outside of the ED [22]. Other research suggests that recovery involves finding one’s identity outside of the ED, including interests, desires, and wants unrelated to weight, shape, or food [16, 30, 55, 62]. Participants in a recent study conducted in our lab reported that this may involve integrating the ED into one’s self-narrative and understanding the role of the ED in shaping identity [21]. This category then, refers to individuals’ (re)discovery of self beyond the ED and may include elements such as interests, motivations, values, or desires.

## External/proximal factors

Outside of the recovery star are external and proximal factors. Individuals with lived experience have described recovery as being context-dependent [21, 35]. This includes external circumstances, such as the COVID-19 pandemic or the death of a loved one. It also includes personal factors which may impact an individual’s capacity to work toward their recovery goals, such as being sick or not sleeping well. It is important to note that external/proximal factors may be changeable or unchangeable. For example, although individuals can have some influence over their own actions, no one can change a global pandemic. In contrast, if recovery is being impacted by poor medication adherence, it is possible to work with individuals to manage medication more effectively.

We have used the term ‘proximal’ to emphasize that these are things that impact people at an individual level, albeit to different degrees. This differs from systems level factors which result in the oppression of one or more groups of people, while other groups experience greater privilege. Having said this, system-level factors will inevitably impact external/proximal factors. For example, systems of white supremacy and anti-Black racism have resulted in beauty stereotypes that typically center European/white features (e.g., straight hair, fair skin). These stereotypes will impact most individuals’ self-perceptions but do not have the same oppressive impact as white supremacy. Thus, white supremacy would be considered the system-level factor, while conventional beauty standards may be considered an external/proximal factor.

Of particular importance, studies have found that societal messages around weight and bodies make it more challenging to engage with and sustain recovery [16, 21, 44, 47, 48]. This diet mentality is the product of anti-fat bias (also referred to as fatphobia) and anti-Black sentiment [63, 64], which are system level factors. We have considered diet culture an external factor, despite its racial and anti-fat origins, because the way it manifests in people’s recovery is not systemic (i.e., it impacts most people in similar ways). Lack of access to diagnosis and treatment because of anti-fat bias and racism would be the systemic variable (described next).

## Systemic factors

At the broadest level of the framework are systemic factors. This circle refers to systems of oppression which may impact and often limit, one’s recovery. Individuals with lived ED experience have shared that recovery is not equally accessible [21], and evidence suggests disparities in diagnosis and treatment [43]. This means that individuals with the most privilege (slim, white, affluent, girls/women in the context of EDs) have greater access to recovery-promoting supports. This is not to say that every individual who fits this profile will recover; the research tells us that this is certainly not true (e.g., [65–67]). Rather these individuals come to the recovery process with advantages over marginalized groups. Limited access to appropriate treatment, limited research/understanding on EDs in diverse populations, and inherent ableism, racism, and anti-fat bias in treatment approaches may all negatively impact an individual’s recovery journey.

Below, we provide a description of how neurodiversity, cis-normativity, and anti-fat bias may play an important role in the recovery process as an example of *some* systemic factors that may impact recovery. Numerous other systems, such as racism and white supremacy, can play a similarly limiting (and intersecting) role(s) in the recovery experience.

### Neurodiversity and autism in recovery

Neurodiversity refers to variation in neurocognitive functioning, including but not limited to autism, attention-deficit hyperactivity disorder (ADHD), Tourette’s syndrome, and schizophrenia [68]. Systems however, are often developed with neurotypical individuals in mind, leaving neurodivergent individuals with unequal access to services [68]. For example, the expectation of higher order executive functioning skills (e.g., planning, organizing, time management) in higher education can limit neurodiverse students’ success [69]. In the context of EDs, there is a burgeoning field of research looking at the experience of autism and EDs. Though just one facet of neurodiversity, we provide a more in-depth overview of this literature as an example of how systems which assume neurotypical functioning may impact recovery efforts.

Rates of autism are significantly higher in EDs, particularly anorexia nervosa, compared to the general public (e.g., [70]). Some have argued that this is a result of self-starvation which mimics some of the traits of autism, such as rigidity and social difficulties [71]; however, evidence suggests that rates of autism are still elevated in individuals no longer engaging in ED symptoms (e.g., [72]) and when individuals are assessed using rigorous assessment tools [73]. Retrospective reports by parents indicate that autism traits are present before the onset of ED behaviours (e.g., [74]). Brede and colleagues [75] argue that autistic traits (e.g., sensory sensitivities, thinking styles) contribute uniquely via direct and indirect pathways to the development of EDs [75].

Despite this, autistic individuals with EDs reported that current services do not acknowledge or address the needs of this unique group [76, 77]. For instance, autistic individuals have shared that commonly assumed motivations for ED behaviours such as fear of weight gain are less relevant. Instead, they shared that motivations related to autism and autistic traits (e.g., sensory sensitivities, social confusion) were more accurate [77]. In a qualitative analysis of lived experience perspectives, Babb and colleagues [76] reported that autistic women with EDs and their parents felt that autism was misunderstood in ED treatment. Specifically, they reported that autistic behaviours (e.g., walking on tiptoes, having food not touch) were interpreted as being driven by the ED, leading patients to be labeled as ‘resistant’ or ‘naughty’ [76]. Even when practitioners were aware of an autism diagnosis, participants felt that autism was not taken into consideration and that they were perceived as ‘too complex’ for services [76]. Autistic individuals are referred to and access a broader range of ED treatment settings compared to their non-autistic peers [78], suggesting that these settings do not know how to manage co-occurring EDs and autism.

Moreover, autistic individuals with EDs have noted several concerns with predominant models of ED treatment. Cognitive behaviour therapy for EDs (CBT-E) is a common treatment approach for adults with EDs [79]. Autistic individuals reported that CBT was not helpful for them because it presumed pre-existing skills that these individuals did not have [76, 78]. For example, one autistic woman described that she was not able to generalize the skills that she had learned because she did not know that this was the goal of treatment [76]. In contrast, more explicit and skills focused approaches, such as dialectical behaviour therapy were viewed as being more useful. Autistic individuals have also shared challenges with group therapy, which is commonly used in ED treatment. Group therapy carries social demands which may make it hard for autistic individuals to engage with treatment and which may be perceived by practitioners as deliberate disengagement [76].

Autistic individuals with EDs also noted concerns related to the physical treatment setting. Many autistic individuals experience sensory sensitivities related and unrelated to food [75]. Bright lights in treatment settings can be aversive to patients, while sounds and smells associated with others eating may be unbearable. Current treatment approaches are limited in their ability to manage these stressors for autistic individuals and suggest the need for adapted services and greater autism education for ED clinicians [76].

Because of these factors, autistic individuals with EDs are disadvantaged when it comes to support for their ED. Although these individuals report being diagnosed at an earlier age, there are few effective treatments for this group and they are often referred from service to service [78]. Expectations of ‘typical’ thinking patterns and social communication may make it hard for individuals to engage with existing programs [76], while inaccurate assumptions about the function and maintenance of ED symptoms often leave individuals feeling misunderstood and unheard [75].

### Cisnormativity in recovery

Cisnormativity refers to the societal expectation or assumption that all people are cisgender. In the ED field, diagnostic criteria and treatment approaches have largely been developed for cis-women [80–82], despite evidence that rates of EDs and ED symptoms are elevated among gender diverse (e.g., trans, non-binary, genderqueer) individuals [80, 83–85]. Moreover, evidence suggests that ED clinicians are ill-equipped to support gender diverse clients and patients [86]. Gender diverse individuals have reported significant negative treatment effects related to gender dysphoria [81].

First, there are several barriers to accessing ED treatment related to cisnormativity. Intake and assessment processes may presume a binary gender identity, and some treatment beds are allocated by gender [81]. Gender diverse individuals may therefore, be deterred from even seeking treatment. Furthermore, gender diverse individuals have shared fears of transphobia in medical settings [81, 86]. Many gender diverse individuals have encountered negative reactions from medical and mental health professionals in the past and may fear stigmatized reactions. Concerns about outing oneself may also come into play [81].

If individuals are able to overcome these barriers, current treatment approaches were not designed with gender diverse individuals in mind and often do not reflect these individuals’ experiences. In particular, gender diverse individuals have cited concerns about assumptions related to etiology (e.g., [81]). Treatment approaches like CBT-E [87] presume that individuals with EDs experience distorted cognitions about body and weight; diet culture and drive for thinness are thought to be central to the development of EDs. Gender diverse individuals report that this is not necessarily true for them and that there are other, gender-specific, factors which come into play in the development and maintenance of an ED [81, 86]. A distinction is made between body dysmorphia, dissatisfaction with one or more parts of one’s body, and gender dysphoria, the feeling that one’s physical body does not match one’s gender identity [81]. Gender diverse individuals share that body image concerns stemming from gender dysphoria relate to the disconnect between body and gender. ED behaviours, such as fasting and exercise, may therefore, serve the purpose of controlling the onset of puberty and development of secondary sex characteristics in adolescence and of achieving a physical body that is more in line with their gender in adulthood [81, 86, 88]. For example, low food intake and body weight may stop menstruation [89].

Because of the distinction between body dysmorphia and gender dysphoria, gender diverse individuals have argued that current body image treatments are insufficient for gender diverse individuals [81, 86]. These individuals suggest that focus on body acceptance can be invalidating and dismissive for gender diverse individuals who feel disconnected from their physical body [86]. Activities like looking at one’s reflection in the mirror for the purpose of desensitization may actually increase anxiety and panic [86]. Body image treatment is more complex in gender diverse individuals and for some people, may involve medical or social transitioning [81, 86].

Gender diverse individuals may therefore, face barriers in accessing support, as well as difficulties when in treatment. Current treatment approaches do not consider gender-related factors, which can lead to feeling isolated or different in treatment [81]. This may then limit these individuals’ ability to benefit from treatment and make gains in their recovery.

### Anti-fat bias in recovery

Anti-fat bias refers to negative beliefs about and attitudes toward people who are perceived to be fat [90]. Kinavey and Cool [91] suggest that this bias may emerge from societal beliefs about weight and health (e.g., thin = healthy, fat = bad) which position weight and health as individualistic, moral imperatives [91]. Research suggests that anti-fat sentiments are present among various healthcare providers, including ED treatment professionals [92]. Kinavey and Cool [91] share examples of anti-fat bias in ED therapy settings, including a ‘client [whose] previous therapist had advised her that, when she is bingeing, she should “picture her arms falling off from diabetic necrosis” to shame herself into stopping’ (pp. 121) [91]. Notably, a recent study found that past experience of anti-fat bias in ED treatment was more predictive of current ED symptoms than internalized anti-fat bias [93], suggesting poorer outcomes for individuals exposed to anti-fat bias in treatment.

Furthermore, anti-fat bias is inherent in many ED treatment modalities. For example, Mulheim and Millner [94] discussed how enhanced cognitive behavioural therapy (CBT-E) sometimes includes reassurances that clinicians do not want to make clients ‘fat,’ just healthy. Though perhaps seemingly benign, such assurances serve to reinforce anti-fat narratives [91]. Similarly, one of the top treatment manuals for binge eating disorder frames fatness in a negative way, encouraging treatment providers to offer advice (e.g., weight management, restriction) that is inconsistent with understandings of binge eating disorder. Moreover, the manual cautions clinicians not to use these same approaches with clients who experience different EDs [95].

Beyond treatment, the diagnosis of EDs is steeped in anti-fat bias. The *Diagnostic and Statistical Manual of Mental Disorders, Fifth Edition Text Revision* (DSM-5-TR) [96] specifies that a diagnosis of anorexia nervosa requires a significantly low body weight and indicators of severity are predicated on BMI. Individuals who are not in the underweight category are given the diagnosis of ‘atypical anorexia nervosa.’ The use of the term ‘atypical’ frames these individuals as deviating from the norm, when in fact atypical anorexia nervosa is much more prevalent than the ‘typical’ form [97]. Despite this, people not viewed as being underweight are less likely to be perceived as fitting the criteria for anorexia nervosa [98]. Among people who do receive a proper diagnosis, fewer sessions are recommended [98] and weight restoration is typically stopped earlier [99] compared to underweight individuals with anorexia nervosa. This occurs despite evidence that individuals with atypical anorexia nervosa experience more extreme ED psychopathology [100].

From the above, it should be apparent how systems of anti-fat bias can limit fat people from making progress in their recovery. First, these individuals are less likely to receive an appropriate diagnosis. If individuals receive an accurate diagnosis, they are often prescribed a different treatment regimen than individuals with similar struggles in smaller bodies. Harrop and colleagues [60] found that while atypical anorexia was more prevalent in epidemiological studies, these individuals were under-represented in treatment settings, suggesting a lack of available treatment. Moreover, if they can access treatment, ‘gold standard’ modalities for treating EDs are inherently anti-fat which further stigmatizes individuals. McDermid [101], a fat activist with lived ED experience, describes the repeated micro-aggressions that are encountered by fat people in ED treatment and how these inevitably fail those individuals. To consider a person’s recovery outside of this context would overlook a significant player in their recovery.

## Implications

There are several clinical, research, and advocacy implications stemming from this framework. We outline these briefly in the next sections. Readers may find more specific recommendations in Table 1.

**Table 1:** Clinical, research, and advocacy implications with specific recommendations

	General implications	Specific examples
Clinical	Inform treatment provider’s assessment and treatment approach	Clinicians may use the recovery framework form in their initial assessment to inform their understanding of treatment goals and external factors which may impact the recovery journey
	Inform treatment targets	Clinicians may talk with clients/patients using the form about what goals they have for treatment, which may inform the treatment modality used
	Inform treatment monitoring	Clinicians may adjust their treatment monitoring according to treatment goals. For example, if someone indicates that self-compassion is important, the clinician may choose to assess self-compassion before session each week
	Inform our own self-assessment	Considering systemic barriers may offer clinicians opportunities to identify areas for growth. For example, a clinician who has less experience with transgender clients may seek out learning opportunities so that they can provide a safe space for these clients
	Suggest the importance of advocacy	Clinicians have a responsibility to advocate for greater representation and inclusion. For example, clinicians may offer sliding scale to marginalized clients. Within formal programs, clinicians may evaluate demographics of clients served and suggest changes to make programs more accessible
Research	Examining acceptability and accuracy of the framework	Researchers may present the framework to individuals with lived experience and solicit their feedback. Qualitative analysis may be used to assess the acceptability of the framework to individuals with lived experience. Focus groups may help to identify any areas that need to be adjusted or removed
	Explore what elements individuals feel are important in recovery	Researchers may explain the framework to individuals and have them fill it out based on their recovery journey. Researchers may then code the content of the recovery star (i.e., what do people include) and/or ask follow-up questions for qualitative analysis
	Evaluate effectiveness of the framework in treatment	Having established the acceptability of the framework, researchers may evaluate the effectiveness of using the framework with clients in treatment using a randomized controlled trial. Participants would be randomized to using the framework or not and then compared on predetermined outcomes
Advocacy	Contextualizing recovery experiences	Advocacy groups and other organizations who ask individuals with lived experience to speak may ask them to contextualize their recovery experience using this framework before sharing their story
	Highlights the need for systemic change	As a field, we need to create a more inclusive and safe space for diverse individuals. Goel et al. [42] provide an excellent set of actionable steps that established and early career professionals can take

### Clinical

**Fig. 2:**
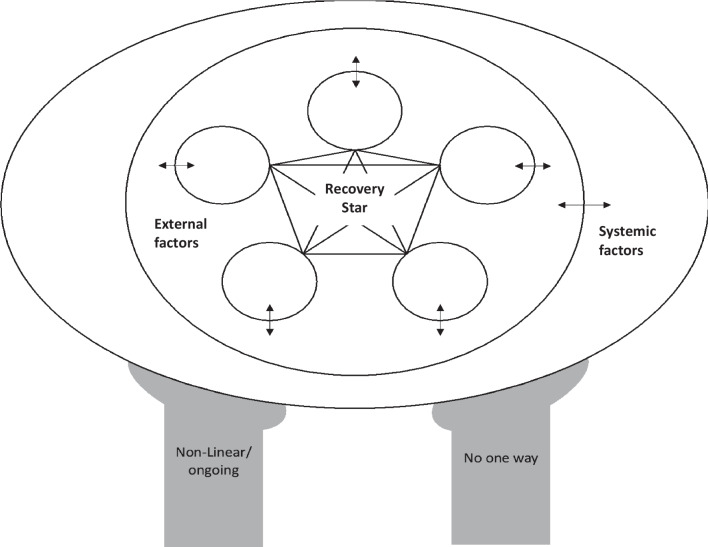
Blank version of the recovery model which can be completed with clients/patients

Clinically, this framework provides a broader understanding of recovery which may inform treatment providers’ assessment and treatment approach. Rather than focusing on narrow outcomes such as weight and observable symptoms, the framework situates an individual’s functioning in their broader life context. Practically, we see this as a tool that can be employed with clients/patients. Figure 2 presents a fillable version of our framework. This can be completed with individuals to better understand their goals for recovery (i.e., outcomes) as well as any potentially limiting factors at the external or systemic level. This offers opportunities to explore which, if any factors, may be changeable in supporting individuals to work toward their desired outcomes. A fictional case study with completed worksheet (S1) is presented in Additional file 1.

Some may argue that this is ill-advised as individuals with EDs may be unlikely to include weight gain and/or behavioural abstinence in their recovery star. We provide two counterpoints. First, as described above, physical restoration and relationship with food, body, and exercise are broader than just weight and stopping behaviours (e.g., [18, 19]). Thus, while an individual may not include ‘weight gain’ per se, they may have elements such as improved energy or ability to think more clearly (as noted by participants in [21]), which still point to some level of physical stability as a desired outcome. Second, and extending upon this, having domains in the recovery star which are incompatible with continued engagement in the ED provide an opportunity for the ED itself to be listed as an external/proximal factor which impacts the recovery star. This then, invites opportunities for motivational interviewing [102] and/or acceptance and commitment therapy [103] techniques which move individuals toward seeing the disconnect between where they want to be and what they are doing. We also point readers to research contending that recovery is not necessarily predicated on behavioural abstinence [104] and re-iterate that given the personal nature of recovery, the role of symptoms and symptom abatement will differ from person to person.

Notably, this framing of recovery highlights the need for social justice work among clinicians. Although typically, psychotherapy is focused at the individual level (see [105] for discussion), our framework situates recovery as influenced by *and* influencing broader contexts. Hence, clinicians have a responsibility to work toward systemic change to support individuals who are less privileged in accessing ED recovery supports, in whatever form they may desire. We suggest that this starts with self-reflection regarding one’s personal biases, as well as the limitations of the field as a whole (e.g., [45]). Kinavey and Cool [91] provide recommendations with respect to anti-fat bias which may be helpful in jump-starting this process.

## Research

Our framework also offers avenues for future research, although this differs from how recovery has been predominantly conceptualized and investigated. As the proposed framework comprises outcomes in concert with process and contextual factors, recovery may not be well suited to quantitative analyses, which tend to result in categorizing individuals as recovered or not. Instead, we suggest that the field may benefit from research examining the inherent complexity of the model and people’s experiences. Inevitably, this invites questions of validity and reliability. Yet, such terms stem from positivist framings, which are antithetical to the proposed approach. Thus, we suggest that a starting point for research would be to examine acceptability and accuracy of the model from the perspective of individuals with lived experience. The goal is not to determine that the model is ‘valid’ but rather that it generally fits the lived experience perspective, across diverse samples of individuals, and to make changes and adaptations as necessary. Given the focus on systemic factors, we believe that it is especially important to solicit views from marginalized individuals. Involving individuals with lived experience in various stages of the research process is paramount to better understand recovery and this model (see [106]).

Having established that the model fits for individuals with lived experience, future research may investigate *what* individuals choose to include as outcomes in their recovery star, as well as which external/proximal and systemic factors may impact the recovery journey. Contrary to current approaches, which focus on statistical associations, we suggest that this offers opportunities for individuals themselves to reflect on the relations between external or systemic factors and their recovery, providing deeper and more nuanced understandings.

There are also opportunities for evaluation of the model in clinical contexts. As discussed, this model may provide a point of discussion for individuals and clinicians. Completing this tool together affords opportunities to better understand desired outcomes and possible limiting factors, and may of course, be subject to evaluation. For example, researchers may be interested in clients’ initial reactions to being presented with the model. Consistent with more traditional approaches, it may be possible to evaluate whether and how the inclusion of this tool impacts long-term outcomes (e.g., quality of life, need for additional treatment).

## Advocacy

Finally, we argue that the proposed framework has implications in recovery advocacy settings. This framework may have utility in contextualizing recovery experiences, which have historically been presented as achievable by everyone. We suggest that framing one’s recovery narrative through this model allows readers/listeners/learners to view recovery as a contextualized phenomenon. It also serves to name the many privileges to which recovery advocates often have access. By presenting recovery in this way, advocates can communicate what supports were available to them and what systems of privilege may have enabled those supports.

This leads into the final, and in our view, most important implication of this framework: the need for systemic change in the ED field (see 45 for description in the context of race). As described with respect to anti-fat bias, assessment, diagnosis, and treatment of EDs is reap with systemic bias. Many of the phenomena involved in EDs were first described by individuals of privilege and not informed by lived experience. This has resulted in a system that caters predominantly to thin, white women of higher economic status. Until systems of oppression have been dismantled, recovery remains an inequitable phenomenon. Studies will continue to be over-represented by thin white women, whose experiences are valid but *not* the only experience of recovery. ED advocacy must involve calls for change at the systems level; otherwise, we continue to perpetuate an inequitable and oppressive system of recovery, which ultimately does everyone harm.

## Conclusion

Recovery in the context of EDs is a nuanced phenomenon. Using various methodological and theoretical approaches, researchers have identified the importance of recovery criteria, as well as external and systemic factors. In this paper, we propose a framework for integrating and expanding these findings. From this perspective, recovery is viewed as an individual and ongoing phenomenon which is impacted by life events and systems of oppression. Considering recovery in this way allows researchers, clinicians, and individuals with lived experience to acknowledge the complexity of and limitations inherent in recovery. This framework may also have merit in advocacy settings and points to the need for greater social justice work in the field to dismantle systems of oppression, which contribute to inequity in recovery.

## Supplementary Information

**Additional file 1**. Fictional case study and completed blank form.

## Data Availability

Not applicable.

## Abbreviations

ED: Eating disorder
CBT: Cognitive behavioural therapy
