# Supplementary material for: More than an outcome: a person-centered, ecological framework for eating disorder recovery
Source: J Eat Disord. 2023 Mar 22;11:45. doi: 10.1186/s40337-023-00768-1 (PMC10032254; doi:10.1186/s40337-023-00768-1)
Supplement: Supplementary file 1 — Additional file 1. Fictional case study and completed blank form. [file 40337_2023_768_MOESM1_ESM.docx]

**Clinical Case Example.** To illustrate the application of the proposed model, we provide a fictional case example with a completed worksheet presented in Figure S1. Alex is a 33-year-old fat genderqueer individual. They presented for therapy at an ED clinic following a referral from their primary care physician. During the assessment, it was determined that Alex struggled primarily with restrictive eating behaviours.

After presenting the framework and introducing the blank form, Alex was invited to think about the areas in which they would like to grow their life (the recovery star). Alex identified five areas for change. First, Alex noted that they wanted to be happy. Having struggled with their gender identity since they were a child, Alex indicated that they had never really experienced happiness or contentment and would like to know what this felt like. Readers may note that ‘happiness’ as described by Alex could fall under multiple categories. First, happiness was added to the recovery star. It was also however, identified that cissexist/cis-normative ideals were impacting Alex’s happiness. Thus, this was added to the systemic factors. Alex identified strongly with this, noting that in addition to cissexism impacting their happiness, they have struggled significantly with getting gender-affirming and ED care since sharing with their doctor that they are genderqueer. When they had been in ED programs in the past, much of the content was focused on female bodies which did not resonate with Alex and left them feeling isolated in treatment. Alex noted that because of this they have become involved with advocacy initiatives focused on promoting awareness of EDs in gender diverse populations. Alex shared that this is an important part of the person they are becoming and as such, advocacy was added to the star.

When asked to elaborate on what feeling happy might include, Alex reported wanting to feel better about their body. Initially, this revolved around losing weight but through discussions, they identified that they just wanted to feel okay in their body. This was labeled body acceptance. They also reported wanting to have more energy. Alex shared that they have been feeling more sluggish and tired lately. This has resulted in leaving events early and struggling to connect with friends, another area of importance to Alex. Both energy and friendships were deemed to be important for Alex’s recovery journey and added to the star.

After completing this part of the form, Alex was invited to reflect on what elements of their environment may be impacting their ability to work toward the elements of the recovery star. Alex shared that they have been struggling with a recent break-up which has prompted some social anxiety. Alex noted that the grief of losing the relationship and the anxiety about being judged has made it hard to connect with other people, which they know can be beneficial for their eating and mood. Alex also shared that many of their colleagues engage in diet talk at work. This impacts how they feel about their body and affects their eating habits throughout the day. When asked about any external factors that might be helpful, Alex shared that after several years they have finally paid off their student loans and are financially stable. They indicated that this will likely be helpful in paying for costs associated with treatment and recovery. They also noted that their family has been supportive both of their gender identity and ED.

Finally, Alex was invited to think more about the systems which may make recovery hard for them. As has already been noted, they identified cissexism as having a detrimental impact on their treatment experiences. They also noted that their weight has been a challenge. Alex elaborated, noting that for several months, their doctor did not believe that they could be struggling with food because of their weight. Moreover, when they expressed concerns related to their gender, it was suggested that they should ‘lose weight to see if that helps.’ These experiences had negatively impacted Alex’s ED and gender journeys. They noted that they had been concerned about returning to treatment for fear that they would be turned away due to their weight. At the same time, Alex was able to acknowledge their privilege as a white individual of middle class.

Looking at Alex’s completed form may offer opportunities for treatment planning. For example, consider that Alex’s difficulty being ‘happy’ occurs in the context of broader cissexist and anti-fat systems. A clinician may therefore, choose to work with Alex on understanding and fighting back against these systems, given Alex’s desire to be an advocate. Without this contextual information, we may be prone to adopting a CBT behavioural activation approach. Though this makes sense in the context of low mood, it puts the onus on the individual to make change when in fact there are legitimate systems which contribute to lower mood and feelings of inadequacy. Considering recovery more broadly and from the perspective of this framework offers opportunities to target more specific and personalized outcomes.

**External factors**

**Systemic factors**

**Recovery Star**

**Figure S1 Completed version of the recovery model for Alex**
